# Targeted Test and Treat at Point of Entry to Reduce Importation of Malaria Parasites: A Systematic Review

**DOI:** 10.4269/ajtmh.22-0771

**Published:** 2023-12-20

**Authors:** Elisabet Martí Coma-Cros, Maria Tusell, Beena Bhamani, Vita Mithi, Elisa Serra-Casas, Nana Aba Williams, Kim A. Lindblade, Koya C. Allen

**Affiliations:** ^1^ Barcelona Institute for Global Health (ISGlobal), Hospital Clínic – University of Barcelona, Barcelona, Spain;; ^2^ Armref Data for Action in Public Health Research Consultancy, Mzuzu, Malawi;; ^3^ Society for Research on Nicotine and Tobacco-Genetics and Omics Network, Madison, Wisconsin;; ^4^ Leaders of Africa Institute, Baltimore, Maryland;; ^5^ Global Malaria Programme, World Health Organization, Geneva, Switzerland

## Abstract

As countries approach malaria elimination, imported cases of malaria make up a larger proportion of all cases and may drive malaria transmission. Targeted test and treat (TTaT) at points of entry (POEs) is a strategy that aims to reduce the number of imported infections in countries approaching elimination by testing and treating individuals at border crossings. No evidence has been systematically collected and evaluated to assess the impact and operational feasibility of this strategy. This systematic review gathered empirical evidence on the effectiveness of the intervention, contextual factors, and results of modeling studies that estimate its potential impact. Bibliographic searches were conducted in March 2021 and updated in April 2022, and a total of 1,569 articles were identified. All study designs were included, but none of them were intervention studies set up to measure the impact of TTaT at POEs. Seven nonrandomized observational studies were eligible for assessment of outcome data in terms of describing the extent of positive cases among people crossing borders. Also included in the review were three studies for assessment of acceptability and feasibility of the intervention and three for assessment of mathematical modeling. The positivity rates reported in the seven studies ranged between 0.0% and 70.0%, which may be attributable to the different settings and operational feasibility. Overall, there is limited evidence of the effect of TTaT at POEs on the prevalence of infection, and the certainty of the evidence was very low owing to critical risk of bias, serious inconsistency, and serious indirectness.

## INTRODUCTION

In 2021, there were 247 million cases and 619,000 deaths from malaria; mortality decreased slightly from 2020.^1^ Vast global efforts and large economic investments have been made worldwide to reduce, control, and eliminate malaria, resulting in a substantial reduction in the burden in the past 20 years.^2^ Nevertheless, this reduction slowed down in 2016 and has further stagnated since the start of the COVID-19 pandemic, leaving malaria as a significant global public health issue.^3^

Malaria importation has a great impact in countries approaching elimination when neighboring countries have sustained malaria transmission.^4^ One key factor that facilitates malaria transmission is the high and constant mobility of populations and vectors between neighboring countries across numerous crossing points and porous national boundaries. In many border areas, residents are considered vulnerable and marginalized populations at high risk of malaria.^5^^,^^6^ In the context of this review, borders or boundaries were considered to be tight or loose depending on the level of security that migrants experienced when crossing the borders.^7^ Other factors can also have an impact on the importation of malaria, such as social (i.e., treatment-seeking behavior or incomplete adherence to medication), political (i.e., conflicts or wars), economic (i.e., lack of resources or access to health services), and environmental (i.e., remoteness of the area or lack of geographical barriers) factors, as well as control interventions implemented in each country such as vector control, case management, and active surveillance.^8^^,^^9^

Some strategies to mitigate malaria importation include cross-border initiatives to mobilize resources where they are most needed or most likely to have the greatest impact,^10^^,^^11^ establishment of functional border coordination, strengthening of surveillance activities for prevention, rapid diagnosis and prompt treatment of malaria cases, and implementation of specific policies and regulations.^4^^,^^9^ Furthermore, interventions and strategies need to be adjusted to the diverse settings and should be targeted to reach specific populations (i.e., adults and men), often in hard to reach locations or in particular contexts (e.g., forest goers, illegal migration, or importation of malaria).^12^^–^^14^

In 2018, the WHO convened an evidence review group (ERG) on border malaria with the objective of summarizing the factors that might influence cross-border malaria and malaria importation, to examine the effectiveness of current tools, and to draw evidence from other global eradication initiatives.^4^ The ERG concluded that there was no universal approach to address border malaria and that countries should consider this problem early to shorten the long tail of elimination.^9^ Still, no evidence has been systematically collected and evaluated to assess the impact of targeted test and treat (TTaT) at points of entry (POEs)^7^ to reduce malaria importation.

The WHO commissioned the Barcelona Institute for Global Health, a WHO collaborating center, to conduct a systematic review of the effectiveness of TTaT at POEs to reduce the importation of malaria transmission, as no recommendations related to this strategy had been developed. With this systematic review, we reviewed all available evidence regarding TTaT at POEs to determine the benefits and harms of testing adults and children with a parasitological test at their POE as they entered or returned to a country or subnational area for work, military service, residence, or tourism or to visit friends and relatives and the treatment of confirmed cases with a full therapeutic course of antimalarial medicine, including radical treatment of *Plasmodium vivax* and *Plasmodium ovale*. In addition, we summarized the evidence on contextual factors including values and preferences, acceptability, health equity, resource use, and feasibility and insights from mathematical modeling studies.

## MATERIALS AND METHODS

### Protocol and registration.

This systematic literature review followed the PRISMA (Preferred Reporting Items for Systematic Reviews and Meta-Analyses)^15^ format, and the protocol was registered in the International Prospective Register of Systematic Reviews, CRD42021240867.^16^ Complete details of the search strategy, eligibility criteria, study selection, data collection, and analysis have been described elsewhere^17^; a brief summary of the methodology is given here.

### Population, intervention, comparison, and outcome (PICO).

This review aimed to answer the following question: “What are the relative effects (benefits and harms) of targeted testing and treatment for malaria at POEs?” Points of entry (i.e., land border crossings, airports, or seaports) could refer to crossings into an area with ongoing malaria transmission that is seeking to eliminate malaria or into an area with malariogenic potential seeking to prevent reestablishment. The PICO question has been described extensively elsewhere.^18^

### Search strategy.

The search strategy for the review was developed in collaboration with a systematic review information specialist and was conducted in March 2021 and updated in April 2022 (Supplemental Table 1).

### Eligibility criteria.

The study characteristics for eligibility have been specified elsewhere.^17^ Both randomized and nonrandomized studies were eligible for inclusion in the review. We excluded studies that did not include at least one of the following outcomes: number of positive cases found as a proportion of total imported cases at the community level, adverse events, prevalence of infection among the targeted group, data on contextual factors, or mathematical modeling results.

### Study selection.

After removing duplicates, four authors (M. T., B. B., V. M., and E. M. C.-C.) from the systematic review team independently reviewed abstracts and titles from the retrieved articles in duplicate. All studies identified as potentially eligible based on the title and abstract reviews were retrieved, and the full report was assessed for eligibility by two authors (M. T. and E. M. C.-C.) independently.

### Data collection.

To minimize errors and bias, two reviewers (M. T. and E. M. C.-C.) extracted crude data including number of cases, number of participants, and any measures with a statistical outcome. Other measures (e.g., adverse events) assessing significance of the outcomes for the targeted population were also extracted independently from the included studies.

### Data synthesis.

Because of the nature of the data reported in the studies, no statistical synthesis could be performed. Considering that the definition for *prevalence* varied across the included studies, the test positivity rate in the targeted populations was calculated by dividing the total number of positive cases by the total number of individuals tested to allow for a comparable measure across studies. This approach facilitated noting of trends in yield or potential impact of the intervention in targeted populations.

### Assessment of risk of bias.

The primary author (E. M. C.-C.) independently assessed the risk of bias in individual studies included in the review, and a secondary author (M. T.) performed an abridged assessment at the domain level for comparison and agreement. For all studies that were considered to be at critical risk of bias, a subsidiary descriptive analysis and a report of their estimates of effect were generated.

## RESULTS

### Summary of selected literature.

A total of 1,569 records were identified: 1,444 via searching databases, 28 from registers, and 97 via other methods (e.g., websites, organizations, and citation searching). Before the screening, 303 records were removed because of duplication, with a total of 1,266 records to be screened against title and abstract eligibility (1,169 identified via databases and registers and 97 identified via other methods). Of these, 65 were assessed for full-text eligibility criteria.

**Figure 1.**
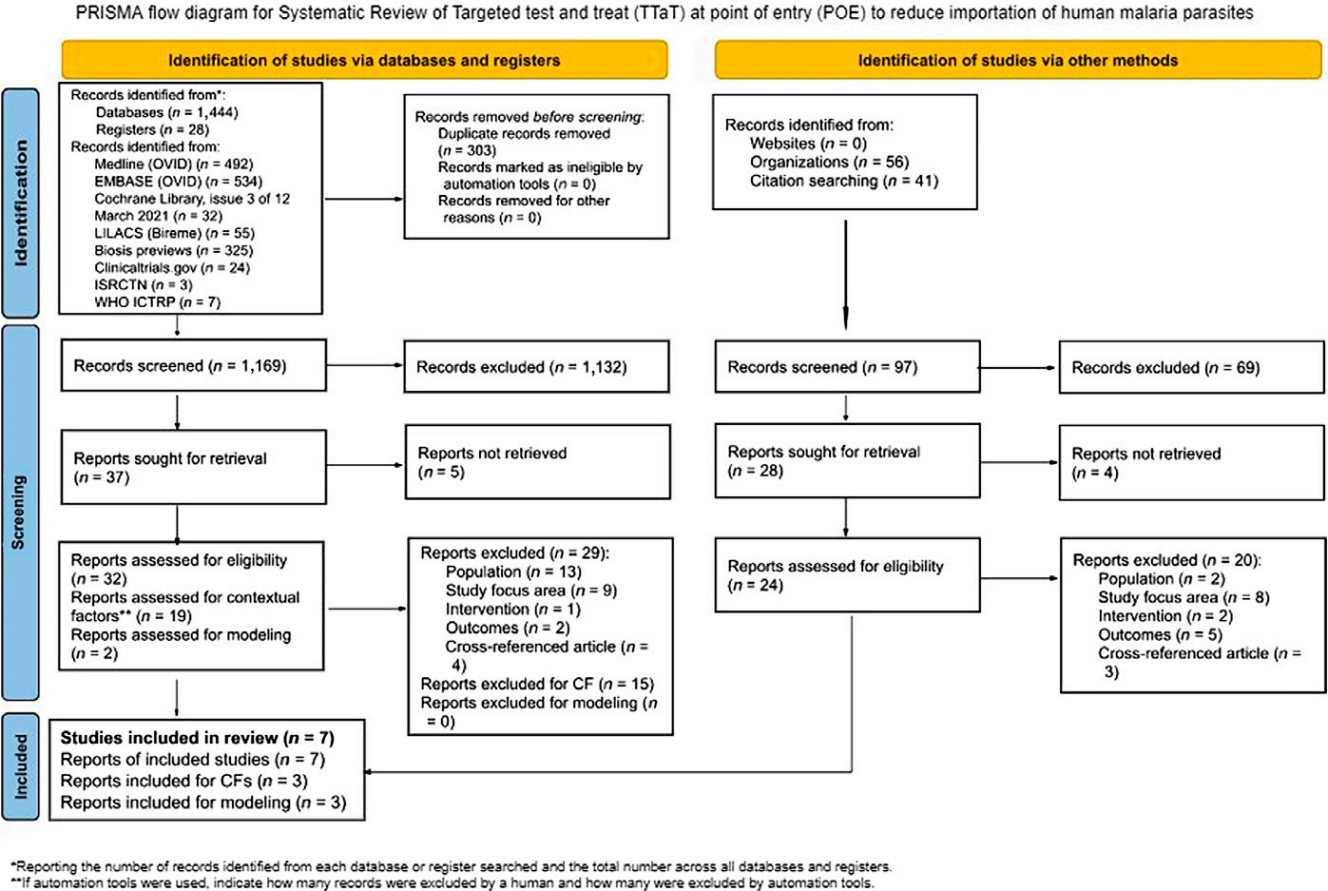
PRISMA flow diagram for systematic review of TTaT at POE to reduce importation of human malaria parasites. CF = contextual factors; ICTRP = International Clinical Trials Registry Platform; ISRCTN = International Standard Randomised Controlled Trial Number; LILACS = Latin American and Caribbean Health Sciences Literature; POE = point of entry; PRISMA = Preferred Reporting Items for Systematic Reviews and Meta-Analyses; TTaT = targeted test and treat.

Fifty-eight articles were excluded after full-text screening for the following reasons: incorrect population (*n =* 15), incorrect intervention (*n =* 3), incorrect study focus area (*n =* 17), cross-referenced article (*n =* 7), incorrect outcome (*n =* 7), or because a full report could not be retrieved (e.g., abstract, protocol, or other document and ongoing studies) (*n =* 9) (Figure 1). All full-text studies that did not meet the eligibility criteria are listed in Supplemental Table 2.

After the full-text screening, seven articles were included for assessment of outcome data in terms of describing the extent of positive cases among people crossing borders; all seven were nonrandomized observational studies conducted in Equatorial Guinea (EG),^19^ the United Arab Emirates (UAE),^20^ Myanmar,^21^ China,^6^ Greece,^22^ and Cambodia.^23^^,^^24^ Descriptive characteristics of the seven included studies are summarized in Table 1 and are described in detail in Supplemental Material 3. Three articles were included for assessment of contextual factors: two articles for acceptability,^23^^,^^25^ and one article for feasibility.^24^ In addition, three articles were included for the assessment of modeling.^26^^–^^28^

**Table 1:** Characteristics of included studies

Study	Location	Year(s)	Study Design	Description	Outcome Reported
Bradley et al.^19^	Bioko Island, EG	2013	Nonrandomized study	Targeted population: passengers traveling by boat from EG mainland to Bioko Island and vice versa Border control: tight Intervention: test and treat Diagnostic tool: RDT Test conditions: on the boat	Prevalence of infection among those targeted by the intervention (reported as prevalence)
Dar et al.^20^	Al Ain District, UAE border area with Oman	1988–1991	Nonrandomized study	Targeted population: all new arrivals applying for resident or work permit Border control: tight Intervention: test and treat Diagnostic tool: microscopy Test conditions: medical checkup	Prevalence of infection among those targeted by the intervention (reported as no. of examined and no. of positive)
Kheang et al.^21^	Myanmar border area with Cambodia and Thailand	2012–2015	Nonrandomized study	Targeted population: migrants entering into or departing from endemic areas Border control: loose Intervention: test and treat (ACD and PCD) Diagnostic tool: fever screening and RDT Test conditions: screening points at key fixed locations	Prevalence of infection among those targeted by the intervention (reported as total tested and positive cases)
Edwards et al.^23^	Cambodia border with Thailand, Laos, and Vietnam	2013–2014	Nonrandomized study	Targeted population: mobile and migrant populations crossing the borders Border control: loose Intervention: test and treat Diagnostic tool: fever screening, RDT, and RT-PCR Test conditions: official border points	Prevalence of infection among those targeted by the intervention (reported as positivity rate)
Stratil et al.^24^	Cambodia border with Thailand, Laos, and Vietnam	2018–2020	Nonrandomized study	Targeted population: remote populations living in forested border areas Border control: loose Intervention: PACD and treat Diagnostic tool: RDT Test conditions: MMPs at border crossings, forest entry points, or marketplaces	Prevalence of infection among those targeted by the intervention (reported as no. of people tested and *Plasmodium falciparum* cases detected)
Li et al.^6^	Shanglin County, China	2013	Nonrandomized study	Targeted population: people returning from overseas malaria-endemic regions, mainly Ghana Border control: tight Intervention: ACD and treat Diagnostic tool: microscopy Test conditions: after entry in the clinic; median interval between return date and diagnosis date was 8 days	Prevalence of infection among those targeted by the intervention (reported as attack rate %)
Tseroni et al.^22^	Evrotas Municipality, Greece	2012–2017	Nonrandomized study	Targeted population: migrants from endemic countries residing in the area and covered by the program Border control: tight Intervention: ACD and treat Diagnostic tool: fever screening, RDT, microscopy, and PCR Test conditions: after entry in the household; median time from arrival to registration ranged from 5 to 90 days (between 2012 and 2017)	Prevalence of infection among those targeted by the intervention (reported as no. of migrants screened and no. of reported malaria cases)

ACD = active case detection; EG = Equatorial Guinea; MMP = mobile malaria post; PACD = proactive case detection; PCD = passive case detection; PCR = polymerase chain reaction; RDT = rapid diagnostic test; RT-PCR = real-time polymerase chain reaction; UAE = United Arab Emirates.

None of the studies included in the review evaluated the impact of the intervention, although all seven studies measured and described malaria prevalence among the targeted group. Bradley et al.^19^ focused on testing by rapid diagnostic test (RDT) of passengers traveling by boat between Bioko Island and the EG mainland. Participants testing positive were referred to a local clinic for appropriate antimalarial treatment. The targeted population in Dar et al.^20^ was all new arrivals applying for resident or work permits in the UAE, mainly the Al Ain District, which shares a border with Oman. Testing was done by microscopy during a medical checkup, and all slide-positive cases were treated with standard chloroquine phosphate at the clinic. Kheang et al.^21^ focused on migrants entering into or departing from endemic areas within Myanmar; the test was done at screening points in key fixed locations in Tanintharyi Region and in Kayin State, by temperature check and RDT. Adequate treatment was offered if necessary. Two related studies^23^^,^^24^ were conducted in the areas of Cambodia bordering Thailand, Laos, and Vietnam in different time periods. The test and treat intervention from Edwards et al.^23^ was implemented between 2013 and 2014 and focused on mobile and migrant populations crossing the borders, with fever screening and RDT done at official border points followed by real-time polymerase chain reaction (RT-PCR). If results were positive, individuals were treated according to Cambodian national treatment guidelines. On the other hand, Stratil et al.^24^ implemented active case detection (ACD) between 2018 and 2020, targeting remote populations living in forested border areas. An RDT test was done in these mobile malaria posts (MMPs) at border crossing areas, at forest entry points, or at marketplaces. Positive cases were treated with artemisinin-based combination therapy (ACT; artesunate-mefloquine with pyronaridine-artesunate) or primaquine. Li et al.^6^ focused on people returning to China from overseas malaria-endemic regions, mainly Ghana, after the government of Ghana began to strictly regulate the gold mining industry. Active malaria screening was done by microscopy (median time interval between return date and diagnosis of 8 days), and treatment was facilitated when necessary: either ACT, chloroquine with primaquine, or chloroquine alone. In Tseroni et al.^22^ the target population was migrants coming from endemic countries and residing in the Evrotas Municipality, Southern Greece, who were covered by the ACD program. Tests were done in migrant households, and the tools used were fever screening, RDT, microscopy, and PCR for confirmation; the median time from arrival to registration was 90, 60, 10, 5, 15, and 7 days in 2012, 2013, 2014, 2015, 2016 and 2017, respectively. Positive cases were treated with directly observed therapy with chloroquine and primaquine (after G6PD testing). The seven studies implemented TTaT at POEs regardless of the type of border they had; four studies^6^^,^^19^^,^^20^^,^^22^ were identified as having a tight border and the other three^21^^,^^23^^,^^24^ had a loose one.

In terms of prevalence or positivity rate reported, Bradley et al.^19^ described prevalence with a 95% CI and *P*-value. Dar et al.^20^ reported the number examined by year and the number of positive cases. Kheang et al.^21^ reported the number of total tested and positive cases, both by fiscal year, and the total malaria positive rate. Edwards et al.^23^ reported the number of tests performed (RDT and RT-PCR), the number of positive cases, and the positivity rate (%) with a 95% CI. Stratil et al.^24^ reported the number of people tested, *Plasmodium* *falciparum* cases detected, and the number of people tested to find one *P. falciparum* case. Li et al.^6^ reported the number of persons screened for malaria, the number of detected malaria infections, and the attack rate (%). Lastly, Tseroni et al.^22^ mentioned the median number (range) of migrants screened during weekly or bimonthly visits and the number of reported malaria cases (imported or locally acquired).

Only Bradley et al.^19^ reported prevalence; the rest of the studies were included even though they did not report prevalence per se, but rather reported other measures that were considered to be estimates for prevalence and therefore relevant to the review. Positivity rates were calculated for all of the studies to enable comparison of results (Table 2).

**Table 2:** Calculated positivity rates for each study by test condition

Study	Test Condition	Positivity Rate (%)
Bradley et al.^19^	Bioko – Mainland/<15 years	38.1
	Mainland – Bioko/<15 years	70.3
	Bioko – Mainland/>15 years	22.6
	Mainland – Bioko/>15 years	35.7
Dar et al.^20^	1988	4.6
	1989	5.2
	1990	6.9
	1991	9.1
Kheang et al.^21^	2012/Screening points	13.1
	2013/Screening points	6.1
	2014/Screening points	3.1
Edwards et al.^23^	Thailand/RDT	0.09
	Vietnam/RDT	1.0
	Laos/RDT	8.0
Stratil et al.^24^	2018/MMPs	9.2
	2019/MMPs	1.1
	2020/MMPs	0.09
Li et al.^6^	Overseas travelers	21.6
Tseroni et al.^22^	2012	1.6
	2013/TDA	0.0
	2014/TDA	0.0
	2015	1.6
	2016	1.4
	2017	1.5

MMP = mobile malaria post; RDT = rapid diagnostic test; TDA = targeted drug administration.

### Results by individual study outcomes.

The highest positivity rate among all studies assessed was 70.3% (71/101) reported in EG^19^ in the trip from the mainland to Bioko Island in passengers younger than 15 years. In the same age range, but from Bioko Island to the mainland, the prevalence was 38.1% (63/165). A similar prevalence, 35.7% (283/793), was found in the opposite direction of the boat trip, but in passengers aged 15 years or older. The lowest prevalence, 22.6% (226/1,000), was found among passengers aged 15 years or older traveling from Bioko Island to the mainland (Supplemental Table 3). A positivity rate of 21.6% (874/4,052) was found in Chinese workers returning from Ghana (Supplemental Table 8).^6^ During the 6-year study from 2012 to 2017 conducted in Greece, positivity rates observed among migrant farmworkers from malaria-endemic countries ranged between 1.4% and 1.6%, except for the years 2013 and 2014 when no malaria cases were identified after implementation of targeted drug administration (TDA)^29^ (Supplemental Table 9).^22^ Dar et al.^20^ identified similar positivity rates during the 4 years of the study in Al Ain District, with a slight increase over time from 4.6% (730/15,732) in 1988 to 5.2% (936/18,022) in 1989, 6.9% (1,282/18,532) in 1990, and 9.1% (1,483/16,317) in 1991 (Supplemental Table 4). A similar range of positivity rates, 13.1% (116/884) in 2012, 6.1% (119/1,953) in 2013, and 3.1% (26/839) in 2014, were identified in the study done in Myanmar,^21^ even though in this case there was a slight decrease over the 3 years of the study (Supplemental Table 5). Two studies^23^^,^^24^ that were conducted in the same borders between Cambodia and Thailand, Cambodia and Laos, and Cambodia and Vietnam showed different trends in positivity rates found by official border points and MMPs, respectively. In Edwards et al.,^23^ the positivity rate found by RDT in border crossers from all nationalities and of all ages between 2013 and 2014 was different depending on the border, being 0.09% (1/1,055) in the border with Thailand, 1.0% (10/1,007) in the border with Vietnam, and 8.0% (92/1,143) in the border with Laos (Supplemental Table 6). In Stratil et al.,^24^ the positivity rates registered by MMPs in all three borders by year varied from 9.2% (545/5,897) in 2018 to 1.1% (136/12,839) in 2019 and 0.09% (12/12,421) in 2020 (Supplemental Table 7).

### Risk of bias assessment.

All nonrandomized observational studies^6^^,^^19^^–^^24^ were assigned a critical overall risk of bias due to study design because they were not designed to measure the impact of TTaT at a POE (Supplementary Material 4). In addition, for certainty of evidence, all of the studies were considered to have serious inconsistencies and serious indirectness (except for Bradley et al.,^19^ which was rated as no serious indirectness). Therefore, as expected owing to the descriptive design of the studies, the certainty of the evidence of the effect of TTaT at POEs on prevalence of infection among the populations targeted by the intervention was graded as very low (Table 3).

**Table 3:** Should testing and treatment at points of entry (border screening) versus no intervention be used for reducing the number of imported cases?

Outcome	No. of Participants (studies)	Certainty	Impact
Prevalence of infection among the group targeted by the intervention (test done at POE)	One observational study^20^	ⴲOOO Very low*	Results indicate that the highest prevalence was in passengers younger than 15 years traveling from the mainland to Bioko (70.3%; 71/101). A lower prevalence was observed for the same age range in the opposite direction (38.1%; 63/165). For passengers older than 15 years, a prevalence of 35.7% (283/793) was observed between the mainland and Bioko and a prevalence of 22.6% (226/1,000) in the opposite direction.
Prevalence (positivity rate) of infection among the group targeted by the intervention (test done at POE)	Four observational studies^21^^,^^22^^,^^24^^,^^25^	ⴲOOO Very low*^,^^†^^‡^	For the UAE, where indigenous cases were zero, importation among arrivals applying for a resident or work permit was between 4.6% (730/15,732) and 9.1% (1,483/16,317) for the study period. In Myanmar, among migrant workers the positivity rate decreased over the years from 13.1% (116/884) to 3.1% (26/839). In Cambodia, official border points identified different positivity rates depending on the neighboring country, being 0.6% (7/1,055) in the border with Thailand, 3.6% (36/1,007) in the border with Vietnam, and 11.5% (131/1,143) in the border with Laos. The MMPs identified a decrease in the positivity rate over the years, from 9.2% (545/5,897) to 0.09% (12/12,421).
Prevalence (positivity rate) of infection among the group targeted by the intervention (test done after entry)	Two observational studies^6^^,^^23^	ⴲOOO Very low*^,^^‡^	Results in Shanglin County, China, showed a positivity rate of 21.6% (874/4,052). Targeted test and treat was conducted in individuals with an overseas travel history, coming mainly from Ghana where they worked in the gold mining sector, within a median of 8 days (range, 0–28 days; interquartile range, 4–18 days) between return date and diagnosis date. Results in Evrotas, Greece, showed a positivity rate of 1.6% (15/920 and 6/384) in 2012 and 2015, 1.4% (12/857) in 2016, and 1.5% (14/934) in 2017. During 2013 and 2014, there were no cases, most probably because TDA was implemented in the area. The median time period from the migrants arriving into Greece to diagnosis date was higher for the years 2012, 2013, and 2014 (90, 60, and 10 days, respectively) than for the years 2015, 2016, and 2017 (5, 15, and 7 days, respectively).

MMP = mobile malaria post; POE = point of entry; TDA = targeted drug administration; UAE = United Arab Emirates.

* Observational study.

^†^ Big differences among positivity rates (from 0.0% to 21.0%).

^‡^ Outcome expressed in positivity rate, not prevalence.

### Contextual factors.

Three articles were assessed for contextual factors: two reported on the acceptability^23^^,^^25^ and one on the feasibility^24^ of TTaT at POEs. One of the studies^23^ on acceptability assessed the number of and reasons for refusals to be tested at border crossing points of Cambodia with Thailand, Vietnam, and Laos. Toward the end of the study period, 22% (904/4,110) of the individuals approached refused to participate. Observational data on the characteristics of the refusal population, including sex, approximate age group, and mode of transport (people in cars were deemed as high socioeconomic status and those on foot as low socioeconomic status), were collected. The main reasons for refusal included not having enough time (51.6%), not perceiving themselves to be at risk of malaria and thus not requiring testing (40.6%), being scared to give blood (34.2%), or having an apparent language or cultural barrier (23.9%).

The second study^25^ was conducted from March to April 2010 and provided information on the feasibility and acceptability of implementing TTaT at POEs, a new approach of surveillance in Isabel Province, Solomon Islands. Focus group discussions, key informant interviews, and informal field observation were used. Participants’ answers suggested high acceptability; however, for it to be effective, RDT coverage would need to be scaled up and prioritized at health facilities. In addition, positive cases should be treated at no cost and followed up. On the other hand, results showed that screening of all travelers entering Isabel Province at ports and airports was not viable because of prevailing financial and logistic constraints.

The feasibility of TTaT at POEs was assessed in Stratil et al.,^24^ who aimed to achieve high testing rates and detecting of *P. falciparum* cases, as well as ensuring constant availability of test and treatment supplies at the Cambodia borders. Different insights such us evidence including testing rates, case clustering, stock information, operational experience, and local knowledge about frequented forest areas and population movements were regularly reviewed and used to continuously adapt the positioning of MMPs, the target locations of outreach activities, the timing of service delivery, and other operational aspects.

### Mathematical modeling data.

Three modeling studies^27^^–^^29^ were identified, all of them based on data from the Mpumalanga Province in South Africa. In the first article,^26^ a deterministic, population-based, nonlinear, ordinary differential equation model fitted to the Mpumalanga malaria data was used to predict the impact of scaling-up of vector control, mass drug administration, a focused mass screen and treat campaign, and foreign source reduction, also known as a reduction of the force of imported infection (alone and in combination). In the second article,^27^ a hybrid metapopulation differential equation and individual based model was developed to simulate the impact of focal screen and treat at the Mpumalanga–Maputo border as a means to decrease the inflow of imported infections. This was the first model designed for this purpose in Mpumalanga, and it predicted that such a campaign, simulated for different levels of resources, coverage, and uptake rates with a variety of screening tools, would not eliminate malaria on its own but could substantially reduce transmission. In the third article,^28^ a metapopulation structure was used to describe movement between five municipalities in the Ehlanzeni District on the eastern border of Mpumalanga and movement between these municipalities and Maputo Province, Mozambique, with a stochastic model. The article concluded that although all strategies (in isolation or in combination) contributed to decreasing local infections, none were able to decrease local infections to zero, mainly because of the continuous stream of imported infections. This highlights the importance of source reduction and a regional approach to malaria control and elimination.

## DISCUSSION

None of the included articles described the impact of TTaT at POEs to reduce malaria importation, but several nonrandomized studies described the prevalence of malaria among people crossing borders as well as the acceptability and feasibility of TTaT at POEs.

The impact of the interventions was difficult or not possible to assess, as none of the included studies followed the targeted population to determine if treatment was effective nor were new malaria cases recorded after receiving the intervention. Furthermore, as expected owing to the scope of the intervention, none of the studies had a controlled or randomized design because mobile populations can hardly be randomly allocated, an intrinsic limitation of these studies. The included studies assessed the prevalence of malaria infection among those targeted by the intervention using different measures such as positivity rate and attack rate. The positivity rates reported in the seven nonrandomized observational studies ranged between 0.0% and 70.0%, and the total number of people tested ranged from 101 to 18,022. The different settings and operational aspects of where the interventions took place may explain the wide differences. In particular, the accessibility of populations and the malaria burden in the country/region of origin were important features among the studies. For instance, the fact that the highest positivity rates were reported by Bradley et al.^19^ could be attributed to EG being a high-transmission area, but also to the feasibility of identifying and targeting passengers when entering or leaving the boat (i.e., a tight boundary). Conversely, the lowest positivity rate was identified in a low-transmission area with loose border control and difficult access to target migrants crossing the border.^24^ Two other studies^21^^,^^24^ showed a decreasing trend in the positivity rate over time, but there was no specific evidence that this was due to the implementation of TTaT at the POE. Therefore, with these results, it was not possible to assess the relative effects (benefits and harms) of TTaT at POEs, rather only to evaluate the yield of the intervention for estimating introduced malaria cases at borders.

Among the seven observational studies included in the review, five^19^^–^^21^^,^^23^^,^^24^ tested travelers at the POE and two^6^^,^^22^ within a specified time frame after entry in clinics or households. The two studies conducting TTaT after entry were included because the average time between arrival and testing was relatively short, making locally acquired infections unlikely. In those areas with a relatively tight and controlled border, travelers were tested on a boat between the mainland and an island,^19^ during a compulsory medical checkup after applying for a resident or work permit,^20^ as part of ACD for people with an overseas travel history,^6^ or in their household.^22^ In areas with a loosely controlled border, testing was done at malaria screening points, either positioned in fixed locations^21^ at official border points^23^ or in MMPs.^24^ It is possible that the implementation of TTaT at POEs had a higher impact in tightly controlled borders, where newcomers are more controlled and surveillance can be easily applied.

Two studies showed the importance of combining targeted strategies to reach high-risk populations for preventing re-establishment of malaria in a certain area. Stratil et al.^24^ attempted to do occasional reactive case detection among co-travelers of index cases, achieving a zero positive rate in 2013 and 2014. Although they were not always feasible or possible, co-traveler investigations tested a total of 1,495 individuals in addition to the 31,157 individuals tested in MMPs. In the second study, Tseroni et al.^22^ implemented TDA during the years 2013 and 2014. There was no overlap in the study populations as TDA targeted migrant residents already living in the area,^29^ whereas TTaT was implemented among new migrant farmworkers. Nevertheless, TDA potentially had an impact on overall transmission, so the trend observed on malaria prevalence during this period was likely not exclusive to TTaT at POEs.

Three studies^23^^–^^25^ contributed data on contextual factors, highlighting the importance of acceptability, feasibility, and sustainability as key determinants when a new malaria control intervention is assessed. Some acceptability enhancers included the distribution of information and educational materials, which raised awareness of malaria among travelers and prepared them to recognize malaria symptoms and seek care rapidly at POEs. The modeling studies^26^^–^^28^ concluded that different strategies in isolation or better in combination (e.g., scale-up of vector control, MDA, focused mass screen and treat campaigns, and reduction of foreign sources) could contribute to decreasing local infections, even though no intervention was able to decrease local infections to zero, mainly because of the continuous stream of imported infections.

Targeted test and treat at a POE can be used to identify “hot border” crossing points and to discriminate them from border points representing less of a threat, allowing for better targeting of surveillance and intervention efforts and optimal use of resources. The location and timing of the screening points, as well as the criteria for screening, were important factors in the malaria positive rates observed. For example, screening points identified a large number of malaria cases by specifically targeting high-risk groups such as mobile populations and migrant workers, sometimes in situations where routine monitoring was not efficient. Likewise, the performance of the diagnostic tool(s) used to detect infections among the target population will have an impact on the effectiveness of the testing procedure in the field. Malaria RDTs are very suitable tools for mobile testing posts, as they allow for parasitological diagnosis at the point-of-care level without the need for laboratory infrastructure or highly skilled health professionals. However, as shown in the study from Edwards et al.,^24^ RDTs failed to detect an important proportion of asymptomatic infections among mobile populations crossing Cambodian borders (compared with RT-PCR results). Thus, the limit of detection of point-of-care tests may not be sufficient to detect all malaria infections in certain epidemiological contexts.

Based on the results of this systematic review and deliberations by the WHO Guideline Development Group, two separate recommendations have been adopted for TTaT at POEs depending on when and where the test is done. The WHO conditionally recommends against TTaT at POEs (land, sea, or air) to reduce importation; however, the WHO conditionally recommends malaria testing and treatment of organized or identifiable groups arriving or returning from malaria-endemic areas. The certainty of evidence for both recommendations was judged to be very low.^30^^,^^31^

To generate evidence to assess TTaT at POEs as an intervention, studies need to be well designed and implemented so that the results can contribute to the evidence base that informs policy. During the literature screening phase of this systematic review, numerous studies on border malaria were identified; however, most of these studies were not interventions implemented at borders to reduce importation. Some studies did test people living in villages located in border areas, but testing did not take into account border crossings and the length of time since their last transit across the border.

Cross-border collaboration and information exchange on the number of imported cases found at the border as a proportion of total imported cases could aid in the generation of evidence for TTaT at POEs as an intervention.^32^^–^^34^ In addition, establishing a follow-up of positive cases (with potential implementation of reactive case detection) could help generate more complete and higher quality data.

Tailored packages of ACD approaches in border POEs in combination with community-based passive case detection in areas frequented by migrants are currently promoted in various subregional elimination strategies such as the Malaria Elimination 8^35^ and the Regional Artemisinin Initiative 3 Elimination (RAI3E).^36^^–^^38^ Strategies such as this may garner evidence to help reevaluate the current recommendation against routine malaria TTaT at POEs. Tracking of ongoing malaria projects using the MESA (Malaria Eradication Scientific Alliance) Track database^39^ of active malaria research can help anticipate emerging evidence and guide planning for updates of this and other malaria-related systematic reviews.

## Supplemental Materials

10.4269/ajtmh.22-0771 Supplemental Materials
