# Supplementary material for: Targeted Test and Treat at Point of Entry to Reduce Importation of Malaria Parasites: A Systematic Review
Source: Am J Trop Med Hyg. 2023 Dec 20;110(4 Suppl):73–81. doi: 10.4269/ajtmh.22-0771 (PMC10993784; doi:10.4269/ajtmh.22-0771)
Supplement: Supplemental Materials [file tpmd220771.SD1.pdf]

Supplementary materials

Targeted Test and Treat at Point of Entry to Reduce Importation of Malaria Parasites: A Systematic Review

Elisabet Martí Coma-Cros[1*], Maria Tusell[1], Beena Bhamani[1], Vita Mithi[2,3,4], Elisa Serra-Casas[1], Nana Aba Williams[1], Kim A. Lindblade[5], Koya C. Allen[1]

[1]Barcelona Institute for Global Health (ISGlobal), Hospital Clínic – University of Barcelona, Barcelona, Spain; [2]Armref Data for Action in Public Health Research Consultancy, Mzuzu, Malawi; [3]Society for Research on Nicotine and Tobacco-Genetics and Omics Network, Madison, USA; [4]Leaders of Africa Institute, Baltimore, USA; [5] Global Malaria Programme, World Health Organization, Geneva, Switzerland

[*] Address correspondence to Elisabet Martí Coma-Cros, Barcelona Institute for Global Health (ISGlobal), Hospital Clínic – University of Barcelona, Carrer del Rosselló 171, Entl. 2a, Barcelona 08036, Spain. E-mail: elisabet.marti@isglobal.org

Supplementary table 1. Search strategy

	Search terms
1	*malaria/
2	exp malaria, falciparum/ or exp malaria, vivax/
3	malaria ovale.mp. or Plasmodium ovale/
4	plasmodium malariae.mp. or Plasmodium malariae/
5	1 or 2 or 3 or 4
6	Antimalarials/
7	Disease Eradication/ or elimination.tw
8	(tailored adj2 (intervention* or treatment* or strateg* or administration)).tw
9	(relapse adj2 prevention).mp
10	Presumptive adj2 (treatment or therapy).tw
11	focal adj2 (drug administration).tw or “focal MDA”.tw
12	targeted adj2 (intervention* or treatment or strateg* or administration).tw
13	6 or 7 or 8 or 9 or 10 or 11 or 12
14	Diagnostic Techniques and Procedures/
15	(screening or screened or diagnosed or diagnostics or test*).tw
16	Point-of-Care Testing/
17	“rapid diagnostic test*” or RDT.tw
18	“parasitological adj2 diagnosis”.tw
19	“Parasitologic adj test*” tw or “symptom screening”.tw
20	“case detection” or PACD or ACD
21	14 or 15 or 16 or 17 or 18 or 19 or 20
22	(borders or frontier* or immigration* or immigrant* or migrant*).tw
23	point of entry.tw or Cross-border.tw
24	“Mobile population*”.tw or “returning worker*”.tw or “mobile worker*”.tw
25	Emigration and Immigration/
26	Border crossing.tw
27	travel*.tw or Travel/
28	22 or 23 or 24 or 25 or 26 or 27
29	5 and 13 and 21 and 28

Supplementary table 2. List of studies excluded after full review and primary reasons for exclusion

Study	Reference	Reason for exclusion
**Bannister-Tyrrell 2019**	1	Incorrect population: not targeted to POE population, targeted to villages.<br>Not the correct outcome reported.
**Betanzos-Reyes 2012**	2	Incorrect population: not targeted to POE population, targeted to a migrants hostal.<br>Incorrect intervention: no treatment mentioned.<br>Not the correct outcome reported.
**Cairo 2017**	3	Abstract only/could not be retrived.<br>Cross-referenced article: Hiwat (2018).
**Canavati 2014**	4	Cross-referenced article: Canavati (2016).
**Carrara 2006**	5	Incorrect population: not targeted to POE population, targeted to health centers.
**Carrara 2013**	6	Incorrect population: not targeted to POE population, targeted to villages.
**Galindo 2019**	7	Cross-referenced article: Galindo (2021) – Malakit Project
**Grueninger 2013**	8	Study focus area.
**Hassanpour 2019**	9,10	Incorrect population: test not done at POE.<br>Incorrect intervention: no treatment mentioned.
**Hiwat 2018**	11	Study focus area – Malakit Project
**Hustedt 2018**	12	Cross-referenced article Stratil (2021).<br>Study focus area.
**Kamolratanakul 1999**	13	Incorrect population: not targeted to POE population, targeted to a district.
**Kasumov 2001**	14	Abstract only/could not be retrieved.
**Kheang 2015**	15	Cross-referenced article: Kheang (2017).
**Krisher 2016**	16	Study focus area.
**Li 2015**	17	Abstract only/could not be retrieved.
**Li 2021**	18	Study focus area.
**Liu 2016**	19	Incorrect intervention: reactive case detection (RCD).
**Lopes 2017**	20	Poster only/could not be retrieved.
**Manning 2018**	21	Protocol only. Cross-referenced article: Lon (2015).
**Moonasar 2016**	22	Study focus area: Commentary article.
**Moonen 2010**	23	Study focus area.
**Mosnier 2020**	24	Incorrect population: not targeted to POE population, targeted to villages.
**Nasir 2020**	25	Study focus area: review.
**O'Sullivan 2011**	26	Not the correct outcome reported.
**Pindolia 2010**	27	Cross-referenced article: Pindolia (2014)
**Raccurt 1997**	28	Abstract only/could not be retrieved.

**Rang 2014**	[29]	Cross-referenced article: Edwards (2015)
**Richards 2009**	[30]	Incorrect population: not targeted to POE population, targeted to villages.<br>Incorrect intervention: Bi-annual screening and treatment of households.
**Rondón-Cotacio 2018**	[31]	Study focus area.
**Malaria Consortium 2021**	[32]	Ongoing study with no results available yet.<br>Project brief only.
**Malaria Elimination 8 2018**	[33]	Project brief only/could not be retrieved.<br>Parental project: Malaria Elimination in Southern Africa (Elimination 8)
**Malaria Consortium 2019**	[34]	Cross-referenced article: Edwards (2015)<br>Cross-referenced article: Lopes (2017)
**Anonymous 2018**	[35]	MESA Track information only.<br>Not the correct outcome reported.
**Anonymous 2017**	[36]	Project brief only/could not be retrieved.
**Anonymous 2009**	[37]	Not the correct outcome reported.
**Anonymous 2015**	[38]	Project brief only/could not be retrieved.
**Silal 2014**	[39]	Study focus area.
**Silal 2015**	[40]	Study focus area.
**Silal 2015**	[41]	Study focus area.
**Villasis 2009**	[42]	Abstract only/could not be retrieved.
**Wangchuk 2019**	[43]	Incorrect intervention: treatment not mentioned for the only positive case found.
**Wangdi 2015**	[44]	Study focus area: book chapter.
**Wangroongsarb 2016**	[45]	Incorrect population: not targeted to POE population, targeted to two sites (rural & urban).
**Wen 2016**	[46]	Study focus area.
**Wickramage 2013**	[47]	Incorrect population: a case study.
**Wiwanitkit 2009**	[48]	Incorrect population: not targeted to POE population, targeted to a rural district.
**Wongsricha 2001**	[49]	Study focus area.
**Xu 2016**	[50]	Incorrect population: not targeted to POE population, targeted to short and long migrants.
**Xu 2021**	[51]	Study focus area: systematic review.
**Yan 2013**	[52]	Incorrect population: not targeted to POE population, targeted to villages.
**Zhang 2016**	[53]	Incorrect population: population not mentioned.<br>Not the correct outcomes reported.
**Zhang 2016**	[54]	Study focus area: positive cases reported from other sources (no intervention).
**Zhou 2014**	[55]	Study focus area: cases reported from the other sources.

**Zhou 2016**	[56]	Incorrect population: not targeted to POE population, targeted to internally displaced persons (IDP) camps and local villages.

Supplementary material 3. Narrative summary of each included study and key main findings

Study 1. Bradley et al.[57]

A key challenge to malaria elimination in Equatorial Guinea (EG) includes the increasing number of travelers between mainland EG and Bioko Island, and therefore, targeted strategies that aid in reducing the malaria burden in passengers arriving from the mainland should be considered to prevent persistent transmission to and from the island. In Bioko Island, there was year-round transmission documented for *P. falciparum* infection in children aged 2-14 years with prevalence rates of 14%, 28% and 18% in 2012, 2013 and 2014, respectively. In the mainland, a prevalence of 59% was reported in 2011 (**Supplementary table 3**). To assess the amount of transmission taking place in a relatively tight border, an observational cross-sectional study was conducted during border crossings via boat sailings from the mainland to the island. Testing and treatment of positive cases were implemented to identify individuals with *P. falciparum* infections during boat crossings once a week within the twice weekly sailings between Bata (Mainland) and Malabo (Bioko Island) for 1 month in December 2013. Passengers were tested using a rapid diagnostic test (RDT) and offered treatment if positive. Bioko has a population of approximately 250000 inhabitants, with approximately 21000 individuals arriving each month from the mainland through the four boat sailings that there are per week. Results showed the highest prevalence in children under 15 years' old that were travelling from the mainland to Bioko (**Supplementary table 3**).

Key findings:

- The study was considered to be at critical risk of bias due to the observational study design.
- The outcome assessed was prevalence among the group targeted by the intervention.
- More positive cases were identified in travelers from the mainland to Bioko and the greatest prevalence rates were identified in children under 15 years of age (**Supplementary table 3**).

- No contextual factors assessed. However, this is a costly approach, as it would entail screen and treat all individuals arriving from the mainland.
- Impact and acceptability need to be evaluated to inform the implementation of targeted interventions such as targeted test and treat (TTaT) at points of entry (POE).

Supplementary table 3. Malaria prevalence in Bioko, Equatorial Guinea, in boat passengers under 15 years old or aged 15 years or more

***P. falciparum* prevalence in passengers travelling from Bioko to the mainland (ML) and vice versa**			
Under 15 years old		Aged 15 years or over	
Direction of travel	**Prevalence**<br>95% CI<br>p-value	Direction of travel	**Prevalence**<br>95% CI<br>p-value
Bioko - ML	38.1% (63)<br>26.1 - 51.2<br>0.017	Bioko - ML	22.6% (226)<br>17.3 - 28.6<br>0.001
ML - Bioko	70.4% (71)<br>58.4 - 80.7<br>0.017	ML - Bioko	35.7% (283)<br>30.1 - 41.6<br>0.001

Study 2. Dar et al[58]

After a concerted effort to reduce importation of malaria and elimination of existing foci in the United Arab Emirates (UAE), a country with tight border control, the relative rate of indigenous malaria was reduced from 20.7% in 1983 to 2.8% in 1988. The vector species in the area are *An. culicifucies* and *An. stephensi*. Even though the inland district of Al Ain was a consolidated area with no local malaria transmission across the undermarked border in Oman by 1993, transmission continued mostly during the transmission season between November and March. In an effort to reduce imported cases, targeted strategies were implemented. A nonrandomized observational study was done to assess test and treat strategies implemented for all new arrivals applying for resident or work permit to identify *P. falciparum*, *P. vivax*, *P. malariae* or mix infections during 4 years, between 1988 to 1991. Testing was done by microscopy and all slide-positive cases of malaria were treated with the standard chloroquine

phosphate dose of 600 mg base. Al Ain district is part of the Abu Dhabi Emirate with a predominantly non-national population of 212000 centered around the city of Al Ain.

Key findings:

- The study was considered to be at critical risk of bias due to the observational study design.
- The outcome assessed was incidence of imported malaria among the group targeted by the intervention in the Al Ain district.
- The number of positive cases (**Supplementary table 4**) remained high enough to enable the introduction of imported malaria into the local *Anopheles spp*. mosquitoes if control measures were relaxed.
- No UAE national acquired the infection in that country.
- The principal sources of malaria were Pakistan and Oman, followed by Sudan and Iran.

Supplementary table 4. Incidence of imported malaria in the Al Ain district, United Arab Emirates (UAE)

Incidence of imported malaria in the Al Ain district, UAE				
Year	1988	1989	1990	1991
Nº examined	15732	18022	18532	16317
Nº positive	730	936	1282	1483
%	5.9%	4.7%	6.9%	9.1%

Study 3. Kheang et al.[59]

A key population to contain the spread of artemisinin resistant (AR) malaria in the border areas between Cambodia, Myanmar and Thailand are mobile populations and migrant workers. Malaria season in Cambodia is between May/June to October and the border control is loose. A nonrandomized observational study was conducted to assess the amount of transmission taking place in these three borders. During the project, village malaria workers (VMWs), mobile malaria clinics (MMCs) and screening points (SPs) were established. SPs, which targeted mobile populations and migrant workers were located in fixed locations, such as bus stations and jetty

terminals. Migrants could voluntarily have their temperature checked, be tested for malaria with a RDT, and receive adequate treatment if necessary between October 2012 and March 2015.

Key findings:

- The study was considered to be at critical risk of bias due to the observational study design.
- The outcome assessed was malaria positive rate among the group targeted by the intervention.
- Although SPs tested a fewer number of people compared to VMWs, they yielded a similar malaria positive rate, 7.10% (4344/116177) and 7.29% (6696/149994), respectively (**Supplementary table 5**).
- The combination of approaches (VMWs, MMCs and SPs) maximized the opportunities for migrants to obtain information and services while they travelled and at arrival to their destination community.
- The location and timing of SPs and the criteria for screening were important factors in the resulting malaria positive rate.
- SPs also raised awareness of malaria among travelers, preparing them to recognize malaria symptoms and seek care more quickly in their destination community.

Supplementary table 5. Malaria positive rate by year and service delivery approach in the border areas with Cambodia, Myanmar and Thailand.

Total tested and positive cases by service delivery approach					
Approach	Year	2012	2013	2014	Total
MMCs	Total tested	41550	61725	66584	169859
	Positive cases	1748	1082	1649	4479
VMWs	Total tested	21978	86316	48754	157048
	Positive cases	3290	5495	2669	11545
**SPs**	Total tested	884	1953	839	3676
	Positive cases	116	119	26	261

**Malaria positive rate by service delivery approach**				
Approach	MMCs	VMWs	**SPs**	Average
Positive rate	2.64%	7.29%	7.10%	4.90%

Study 4. Edwards et al.[60]

According to the Cambodia Malaria Survey 2010, prevalence of malaria in Cambodia in 2010 had fallen greatly to only 0.9% in the general population and was concentrated in certain high risk groups such as mobile populations and forest-goers (1.5% and 2.5% respectively). In response to the need to document the spread of malaria across borders, this nonrandomized observational study aimed to quantify the extent of malaria infection, including asymptomatic infection and AR parasites, in border crossing populations at specific sites on each of the Cambodian borders with Thailand, Laos and Vietnam, and to potentially identify "hot borders" in the country, even though control is very loose. From mid-August 2013 until mid-February 2014, booths were set up at each of the three selected official border points. Sample size was calculated at 3000 (e.g. 1000 individuals per site), sufficient to provide enough precision (alpha = 0.05, power = 0.9) to capture at least a 3% prevalence. Any person (of all ages) crossing the border was eligible for participation. Participant temperature was taken using an infrared thermometer in order to test for presence of fever (temperature of 37.5°C). Malaria diagnosis was determined by the RDT SD BIOLINE Malaria Ag P.f/P.v (Standard Diagnostics). If positive, they were treated according to Cambodian national treatment guidelines.

Key findings:

- The study was considered to be at critical risk of bias due to the observational study design.
- The outcome assessed was positivity rate (95% CI) among the group targeted by the intervention.
- Between August 2013 and March 2014, there was a positivity rate of 3.2% (2.6 – 3.8) (**Supplementary table 6**).
- A very high proportion of *Plasmodium* infections were asymptomatic.
- By real time-PCR, the average positive rate identified was 5.4% (4.6 – 6.2), and a high proportion of asymptomatic cases, around 70%, were identified. By RDT the positive rate

decreased to 3.2% (2.6 – 3.8) showing a low sensitivity of the RDT compared to real time – polymerase chain reaction (RT-PCR) (**Supplementary table 6**).

- The high proportion of AR *P. falciparum* infections observed was particularly worrisome, as Laos was not formally acknowledged as affected by confirmed AR at the time of the study.

Supplementary table 6. Malaria prevalence and proportion of positive cases found by RDT and RT-PCR in Cambodia in the border areas with Laos, Thailand and Vietnam.

Malaria prevalence evaluated by microscopy in Cambodia in 2010		
General population	High risk groups<br>Mobile populations – Forest-goers	
0.9%	1.5%	2.8%

**Proportion of sampled individuals found to have *Plasmodium* infection by RDT**				
	Thailand	Vietnam	Laos	Total
Nº of tested	1055	1007	1144	3206
Positive cases	1	10	92	103
Positivity rate % (95% CI)	0.1 (0 - 1.0)	1.0 (0.4 - 1.6)	8.0 (6.5 - 9.6)	3.2 (2.6 – 3.8)

**Proportion of sampled individuals found to have *Plasmodium* infection by RT-PCR**				
	Thailand	Vietnam	Laos	Total
Nº of tested	1055	1007	1143	3205
Positive cases	7	36	131	174
Positivity rate % (95% CI)	0.7 (0.2 - 1.2)	3.6 (2.4 - 4.7)	11.5 (9.6 - 13.3)	5.4 (4.6 – 6.2)

Study 5. Stratil et al.[61]

A key element of the Regional Artemisinin-resistance Initiative Two Elimination (RAI2E) is tailoring a package of active case detection (ACD) and expanding access to early diagnosis and effective treatment. A nonrandomized observational study was conducted to assess transmission taking place among remote populations in border areas with Laos, Thailand and Vietnam where the control is loose between January 2018 and December 2020. Pro-active case detection (PACD) was delivered through mobile malaria posts (MMPs) and outreach activities,

and occasional reactive case detection (RCD) among co-travellers of index cases when feasible. Mobile malaria workers (MMWs) were asked to conduct RCD if a confirmed malaria case had spent time in the forest in the previous two weeks. MMPs were placed at border crossings, at forest entry points or market places. MMPs operated seven days per week with standard operating hours between 7 am and 7 pm with operational flexibility to adjust to local population movement patterns. Everyone that passed their post was eligible for RDT testing. Malaria tests were conducted with *P. falciparum*/*P. vivax* RDTs (SD BIOLINE Malaria Ag P.f/P.v, Standard Diagnostics) without prior screening for fever. Uncomplicated *P. falciparum*, mixed and *P. vivax* cases were treated with artemisinin-based combination therapy (ACT). First-line ACT was artesunate-mefloquine with pyronaridine-artesunate used only during prolonged stock-out of first line treatment. Single low-dose primaquine was given to *P. falciparum* and mixed cases.

Key findings:

- The study was considered to be at critical risk of bias due to the observational study design.
- The outcome assessed was number of tested and number of positive cases among the group targeted by the intervention.
- MMWs under this project contributed to testing 45% (80988/180732) of all people tested in the study and detected 39% (1280/3243) of all *P. falciparum* cases and 72% (1280/1768) of community *P. falciparum* cases (VMW and MMWs) between 2018 and 2020 (**Supplementary table 7**).
- The results presented a successful approach to implementing tailored ACD strategies. Key components of the project success were a combination of proactive and RCD activities tailored to the local target population and context, operational flexibility, strong relationships with local communities, close supervision and quality assurance of service delivery and responsive systems that were able to adapt to changing circumstances.

Supplementary table 7. Malaria positive rate by year and by case detection approach in Cambodia in the border areas with Laos, Thailand and Vietnam.

Total tested and positive cases by case detection approach					
Approach	Year	2018	2019	2020	Total
MMPs	Total tested	5897	12839	12421	31157
	Positive cases	545	136	12	693
Outreach	Total tested	6906	19839	21389	47988
	Positive cases	343	170	20	533
Co-travellers	Total tested	604	768	123	1495
	Positive cases	46	8	-	54

**Malaria positive rate by case detection approach**				
Approach	MMPs	Outreach	Co-travellers	Average
Positive rate	1.57%	1.11%	3.61%	2.09%

Study 6. Li et al.[62]

The incidence of malaria in China decreased sharply to 0.18 cases per 100000 persons in 2012. However, imported malaria among persons returning from overseas malaria-endemic regions was a documented challenge for malaria elimination. For example, in the Shanglin County there was a large outbreak of imported malaria among Chinese workers returning from overseas countries, mainly Ghana (vector species in the area *An. sinensis*). As a result, a nonrandomized observational study was conducted to analyze active malaria screening during May 1 – August 31, 2013, for people with an overseas travel history during the previous years. Testing was not done at POE, but since China has a tight border control, it permitted the median interval time between return date and diagnosis date to be eight days (range 0–28 days; interquartile range 4–18 days). Diagnosis was done by microscopy and all positive cases with *P. falciparum* infection were treated with ACT; persons who had no glucose-6-phosphate dehydrogenase deficiency and who were infected with *P. vivax* or *P. ovale* were radically cured with chloroquine combined with primaquine; and persons who had *P. malariae* infection were treated with chloroquine.

Key findings:

- The study was considered to be at critical risk of bias due to the observational study design.
- The outcome assessed was attack rate among the group targeted by the intervention.
- During the study period there was an attack rate of 21.6% (**Supplementary table 8**).
- Considering the remarkably increasing volume of cross-border travel, malaria imported from overseas countries was a new challenge for malaria elimination in China at the time of the study.
- Measures to prevent mosquito bites and chemoprophylaxis should be addressed to groups at high occupational risk for malaria.

Supplementary table 8. Attack rate and *Plasmodium* spp. for positive cases in the Shanglin County, China.

**Results of attack rate in persons with overseas travel**	
Nº persons screened for malaria	4052
Nº detected malaria infection	874
Attack rate (%)	21.6
Plasmodium spp. for the positive cases	
P. falciparum	827
P. vivax	42
P. malariae	1
P. ovale	1
Plasmodium spp. co-infection (Pf/Pv)	3

Study 7. Tseroni et al.[63]

Greece became a malaria-free country in 1974, but since 2009, imported *P. vivax* cases have been reported. In 2011, an outbreak of 36 *P. vivax* locally acquired malaria cases occurred in the Evrotas Municipality in the Peloponnese, southern Greece, a historical malaria hotspot with a tight border control. The vector species in the area was *An. sacharovi*. To interrupt local malaria

transmission and avoid reintroduction, a PACD program was implemented from 2012 to 2017 in combination with other vector control interventions and targeted drug administration (TDA) during 2013 and 2014 to all migrants from endemic countries residing in the specific area. To assess the ACD program an observational study was conducted. Testing was done in households by the field team, who screened for fever and other malaria compatible symptoms, and tested every suspected malaria case. For suspected malaria cases an RDT (with a *P. vivax* panel detection score of 91.4% at 200 parasites/µL) and/or blood sampling was performed. In the event of a positive RDT, the patient was treated with directly observed therapy (DOT) with chloroquine and primaquine, and further monitored. The median time period between the arrival of the migrants to Greece and the day of their first contact with the field team was much higher for the years 2012–2014 (90, 60 and 10 days respectively), compared with the years 2015–2017 (5, 15 and 7 days respectively).

Key findings:

- The study was considered to be at critical risk of bias due to the observational study design.
- The outcome assessed was number of tested and number of positive cases among the group targeted by the intervention.
- The program recorded a limited number of sporadic introduced cases and a steadily increasing annual number of imported *P. vivax* malaria cases in 2013–2017 (**Supplementary table 9**).
- The study population originated mainly from Pakistan, where *P. vivax* prevalence ranges from 2.4% in Punjab province to 10.8% in Sindh province.
- No contextual factors assessed but the study showed that being undocumented usually makes migrants hesitant in their approach to health care services and that they welcomed treatment at home.

- Surveillance indicators improved significantly over the years as no locally acquired malaria cases were reported in 2016–2017, even though the number of newly incoming migrants increased and no TDA was performed.
- Although the PACD program in Evrotas contributed to the reduction of disease transmission in the area after the cluster peak of 2011 due to the implementation of multiple public health interventions, it was not feasible to accurately estimate the impact of each intervention.

Supplementary table 9. Number of migrants screened, number of positive cases and median time in days from arrival to registration in Evrotas Municipality, Greece.

**Number of migrants screened and number of positive cases in Evrotas Municipality, 2012 - 2017**				
Project year	Median nº of migrants screened	Nº of reported malaria cases	Nº of malaria cases among migrants detected through PACD	Median time (range) from arrival to registration, days
2012	920	17	15	90
2013	582	0	0	60
2014	496	0	0	10
2015	384	7	6	5
2016	857	15	12	15
2017	934	14	14	7
**TOTAL**	4173	53	47	-

Supplementary material 4. Risk of bias assessment:

Study 1. Bradley et al.[57]

Risk of bias
Outcome: **Prevalence of infection**
**Justification**
"The study uses observational data. Although many observed confounding variables were adjusted for in this analysis, there could still be residual confounding"
"This study is likely to have underestimated the proportion of Bioko residents who travelled to mainland EG."
Critical overall risk of bias due to the inherent biases associated with the study design.

Study 2. Dar et al[58]

Risk of bias
Outcome: **Prevalence of infection**
**Justification**
** No bias mentioned in the article.*
Critical overall risk of bias due to the inherent biases associated with the study design.

Study 3. Kheang et al.[59]

Risk of bias
Outcome: **Prevalence of infection**
**Justification**
** No bias mentioned in the article.*
Critical overall risk of bias due to the inherent biases associated with the study design.

Study 4. Edwards et al.[60]

Risk of bias
Outcome: **Prevalence of infection**
**Justification**
"Border points were not selected at random, they were chosen in order to be viable for logistic and operational reasons."
"It was also observed that there was a population of border crossers unable to be approached at all. These were verbally reported by field teams to predominantly be people travelling in cars, trucks and buses, and thus assumedly of a higher SES compared to those crossing on foot."<br>"There was also a selection bias in the study population (where participants were predominantly Cambodians) and was likely observed due to the fact of having the screening booths on the Cambodian side of the border"
Critical overall risk of bias due to the inherent biases associated with the study design.

Study 5. Stratil et al.[61]

Risk of bias
Outcome: **Prevalence of infection**
**Justification**

“As the size of target population had not been formally quantified, this case study could not determine changes in malaria incidence among the target population over time.”
Critical overall risk of bias due to the inherent biases associated with the study design.

Study 6. Li et al.[62]

**Risk of bias**
Outcome: **Prevalence of infection**
**Justification**
“Chemoprophylaxis and detailed exposure history in Ghana were not well documented because most returning miners lacked knowledge and awareness of malaria. In addition, recall was likely to have been poor, given that the miners had lived and worked overseas for a long time at the time of investigation”<br>Critical overall risk of bias due to the inherent biases associated with the study design.

Study 7. Tseroni et al.[63]

**Risk of bias**
Outcome: **Prevalence of infection**
**Justification**
“A mobile population constitutes a limitation for the success of a PACD program; however, we developed several procedures to overcome the relevant challenges. We designed processes for locating, registration, verification and follow up for each migrant staying in the area for more than 24 h, leading to the development of our ACD databases and procedures.”
Critical overall risk of bias due to the inherent biases associated with the study design.

References:

1. Bannister-Tyrrell M, et al., 2019. Forest Goers and Multidrug-Resistant Malaria in Cambodia: An Ethnographic Study. *Am J Trop Med Hyg 100*: 1170–1178

2. Betanzos-Reyes ÁF, González-Cerón L, Rodríguez MH, Torres-Monzón JA., 2012. Seroepidemiología del paludismo en un grupo de migrantes en tránsito (Chiapas, 2008). *Salud Publica Mex 54*: 523–529

3. Cairo H, Hardjopawiro L, Hiwat H., 2017. Migration as a determinant of malaria in Suriname: Challenges in reaching elimination. *ASTMH*

4. Canavati de la Torre SE, Chea N, Whittaker M., 2014. How to implement prevention, test, treat and track (P+T3) amongst populations at higher risk of malaria: lessons from western Cambodia. *13th International Congress of Parasitology (ICOPA)*

5. Carrara VI, et al., 2006. Deployment of Early Diagnosis and Mefloquine- Artesunate Treatment of Falciparum Malaria in Thailand: The Tak Malaria Initiative. *PLoS Med 3*: e183

6. Carrara VI, et al., 2013. Malaria Burden and Artemisinin Resistance in the Mobile and Migrant Population on the Thai–Myanmar Border, 1999–2011: An Observational Study. *PLoS Med 10*: e1001398

7. Galindo MS, et al., 2019. Experience from the field: How to implement a complex intervention study to control malaria in remote areas of the Amazon forest, within a cross-border context? *ASTMH2*

8. Grueninger H, Hamed K., 2013. Transitioning from malaria control to elimination: the vital role of ACTs. *Trends Parasitol 29*: 60–64

9. Hassanpour G, Mohebali M, Mirhendi H, Zeraati H, Raeisi A, Noohi M, Keshavarz H., 2017. A cohort study on asymptomatic Plasmodium-infected malaria among immigrants in malaria--elimination programmed areas of south eastern Iran to underpin malaria eradication. *4th International Conference on Parasitology*

10. Hassanpour G, Mohebali M, Mirhendi H, Zeraati H, Raeisi A, Keshavarz H., 2019. Asymptomatic malaria infections among immigrants in malaria-elimination programmed

areas of south eastern Iran may threaten malaria eradication. *Travel Med Infect Dis 31*: 101426

11. Hiwat H, Martínez-López B, Cairo H, Hardjopawiro L, Boerleider A, Duarte EC, Yadon ZE., 2018. Malaria epidemiology in Suriname from 2000 to 2016: trends, opportunities and challenges for elimination. *Malar J 17*: 418

12. Hustedt J, et al., 2018. Expanding the health system into the forest: Analysis of and response to the challenge of providing malaria services inside forest areas within the Greater Mekong Subregion in the context of malaria elimination. *ASTMH*

13. Kamolratanakul P, Butraporn P, Prasittisuk C, Prasittisuk M, Indaratna K., 1999. Cost and performance of malaria sector: a case study at Malaria Sector 11, Tak Province, Thailand. *Southeast Asian J Trop Med Public Health 30*: 421–6

14. Kasumov VK, Guseĭnov FZ., 2001. [Current malaria situation in Azerbaijan]. *Med Parazitol (Mosk)*: 18–9

15. Kheang ST., 2015. Increasing accessibility of malaria services among cross-border populations in the Greater Mekong sub-region. *ASTMH*

16. Krisher LK, et al., 2016. Successful malaria elimination in the Ecuador–Peru border region: epidemiology and lessons learned. *Malar J 15*: 573

17. Li S, Wang J, Yin S, Li X, Feng X., 2015. [Malaria surveillance in Tengchong County of Yunnan Province in 2013]. *Zhongguo Xue Xi Chong Bing Fang Zhi Za Zhi 27*: 520–2

18. Li X-H, Zhou H-N, Xu J-W, Lin Z-R, Sun X-D, Li J-Y, Lin X-X, Xie Y, Alonso P, Yang H-L., 2021. Seven decades towards malaria elimination in Yunnan, China. *Malar J 20*: 147

19. Liu H, et al., 2016. Investigation and control of a Plasmodium falciparum malaria outbreak in Shan Special Region II of Myanmar along the China-Myanmar Border from June to December 2014. *Infect Dis Poverty 5*: 32

20. Lopes S, et al., 2017. Cross border surveillance initiatives targeting mobile and migrant populations: lessons learnt from Cambodia. *Keystone Symposia Global Health Series on "Malaria: From Innovation to Eradication"*

21. Manning J, et al., 2018. Cluster-randomized trial of monthly malaria prophylaxis versus

focused screening and treatment: a study protocol to define malaria elimination strategies in Cambodia. *Trials 19*: 558

22. Moonasar D, Maharaj R, Kunene S, Candrinho B, Saute F, Ntshalintshali N, Morris N., 2016. Towards malaria elimination in the MOSASWA (Mozambique, South Africa and Swaziland) region. *Malar J 15*: 419

23. Moonen B, et al., 2010. Operational strategies to achieve and maintain malaria elimination. *Lancet 376*: 1592–1603

24. Mosnier E, et al., 2020. Resurgence risk for malaria, and the characterization of a recent outbreak in an Amazonian border area between French Guiana and Brazil. *BMC Infect Dis 20*: 373

25. Nasir SMI, Amarasekara S, Wickremasinghe R, Fernando D, Udagama P., 2020. Prevention of re-establishment of malaria: historical perspective and future prospects. *Malar J 19*: 452

26. O'Sullivan M, Kenilorea G, Yamaguchi Y, Bobogare A, Losi L, Atkinson J-A, Vallely A, Whittaker M, Tanner M, Wijesinghe R., 2011. Malaria elimination in Isabel Province, Solomon Islands: establishing a surveillance-response system to prevent introduction and reintroduction of malaria. *Malar J 10*: 235

27. Pindolia DK, Garcia AJ, Huang Z, Fik T, Smith DL, Tatem AJ., 2010. Quantifying cross-border movements and migrations for guiding the strategic planning of malaria control and elimination. *Malar J 13*: 169

28. Raccurt CP., 1997. [Malaria, anopheles, the anti-malaria campaign in French Guyana: between dogmatism and judgment]. *Med Trop (Mars) 57*: 401–6

29. Rang C, Edwards H, Uth S, Canavati S, Van H, Siv S, Po L, Hustedt J, Roca-Feltrer A., 2014. Identification of asymptomatic malaria infection in border crossing populations in Cambodia. *ASTMH*

30. Richards AK, Banek K, Mullany LC, Lee CI, Smith L, Oo EKS, Lee TJ., 2009. Cross-border malaria control for internally displaced persons: observational results from a pilot programme in eastern Burma/Myanmar. *Trop Med Int Heal 14*: 512–521

31. Rondón-Cotacio M, Tobón-Castaño A., 2018. Actividades de vigilancia epidemiológica de

la malaria en la red diagnóstica de la frontera colombiana-peruana. *Rev Peru Med Exp Salud Publica 35*: 373

32. Malaria Consortium., 2021. *Expanding mobile malaria services to hard-to-reach communities in northern Cambodia*

33. Malaria Elimination 8., 2018. *Impact of the Elimination 8 Driving Collaboration and Accelerating Progress Toward a Malaria-Free Southern Africa*

34. Malaria Consortium., 2019. Accelerating malaria elimination in northern Cambodia through early diagnosis and treatment

35. MESA Malaria., 2018. Extending effective malaria control and artemisinin resistance containment activities to hard-to-reach and transient populations in priority areas of Southeast Myanmar. Available at: https://mesamalaria.org/mesa-track/extending-effective-malaria-control-and-artemisinin-resistance-containment-activities. Accessed. 2018

36. MESA Malaria., 2017. E8 Border Malaria Post Impact Evaluation. Available at: http://mesamalaria.org/mesa-track/e8-border-malaria-post-impact-evaluation. Accessed. 2017

37. MESA Malaria., 2009. Malaria Elimination in Southern Africa (Elimination 8). Available at: http://mesamalaria.org/mesa-track/malaria-elimination-southern-africa-elimination-8. Accessed. 2009

38. MESA Malaria., 2015. Enhanced responses with various approaches to Active Case Detection: Re-Active case detection and investigation of Unofficial Border Crossings. Available at: http://mesamalaria.org/mesa-track/enhanced-responses-various-approaches-active-case-detection-re-active-case-detection-and. Accessed. 2015

39. Silal SP, Little F, Barnes KI, White LJ., 2014. Towards malaria elimination in Mpumalanga, South Africa: a population-level mathematical modelling approach. *Malar J 13*: 297

40. Silal SP, Little F, Barnes KI, White LJ., 2015. Predicting the impact of border control on malaria transmission: a simulated focal screen and treat campaign. *Malar J 14*: 268

41. Silal SP, Little F, Barnes KI, White LJ., 2015. Hitting a Moving Target: A Model for Malaria Elimination in the Presence of Population Movement. *PLoS One 10*: e0144990

42. Villasis E, Cacho J, Bendezu J, Neyra V, Bernal JM., Gamboa D., 2009. Asymptomatic malaria detection by PCR among a collateral native population in an endemic region at the Peruvian-Ecuadorian border. *ASTMH*

43. Wangchuk S, Gyeltshen S, Dorji K, Wangdi T, Dukpa T, Namgay R, Dorjee S, Tobgay T, Chaijaroenkul W, Na-Bangchang K., 2019. Malaria elimination in Bhutan: asymptomatic malaria cases in the Bhutanese population living in malaria-risk areas and in migrant workers from India. *Rev Inst Med Trop Sao Paulo 61*

44. Wangdi K, Gatton ML, Kelly GC, Clements AC., 2015. Cross-Border Malaria: A Major Obstacle for Malaria Elimination. 79–107

45. Wangroongsarb P, et al., 2016. Using Respondent Driven Sampling to Identify Malaria Risks and Occupational Networks among Migrant Workers in Ranong, Thailand. *PLoS One 11*: e0168371

46. Wen S, et al., 2016. Targeting populations at higher risk for malaria: a survey of national malaria elimination programmes in the Asia Pacific. *Malar J 15*: 271

47. Wickramage K, Galappaththy GNL, Dayarathne D, Peiris SL, Basnayake RN, Mosca D, Jacobs J., 2013. Irregular Migration as a Potential Source of Malaria Reintroduction in Sri Lanka and Use of Malaria Rapid Diagnostic Tests at Point-of-Entry Screening. *Case Rep Med 2013*: 1–4

48. Wiwanitkit V., 2002. High Prevalence of Malaria in Myanmar Migrant Workers in a Rural District Near the Thailand-Myanmar Border. *Scand J Infect Dis 34*: 236–237

49. Wongsrichanalai C, Sirichaisinthop J, Karwacki JJ, Congpuong K, Miller RS, Pang L, Thimasarn K., 2001. Drug resistant malaria on the Thai-Myanmar and Thai-Cambodian borders. *Southeast Asian J Trop Med Public Health 32*: 41–9

50. Xu J-W, et al., 2016. Malaria control along China-Myanmar Border during 2007–2013: an integrated impact evaluation. *Infect Dis Poverty 5*: 75

51. Xu J-W, Lee R, Li X-H, Liu H., 2021. Transition of radical, preventive and presumptive treatment regimens for malaria in China: a systematic review. *Malar J 20*: 10

52. Yan J, et al., 2013. Performance of two rapid diagnostic tests for malaria diagnosis at the China-Myanmar border area. *Malar J 12*: 73

53. Zhang J, Dong J-Q, Li J-Y, Zhang Y, Tian Y-H, Sun X-Y, Zhang G-Y, Li Q-P, Xu X-Y, Cai T., 2016. Effectiveness and impact of the cross-border healthcare model as implemented by non-governmental organizations: case study of the malaria control programs by health poverty action on the China-Myanmar border. *Infect Dis Poverty 5*: 80

54. Zhang Q, Sun J, Zhang Z, Geng Q, Lai S, Hu W, Clements ACA, Li Z., 2016. Risk assessment of malaria in land border regions of China in the context of malaria elimination. *Malar J 15*: 546

55. Zhou G, et al., 2014. Clinical Malaria along the China–Myanmar Border, Yunnan Province, China, January 2011–August 2012. *Emerg Infect Dis 20*: 681–684

56. Zhou G, Lo E, Zhong D, Wang X, Wang Y, Malla S, Lee M, Yang Z, Cui L, Yan G., 2016. Impact of interventions on malaria in internally displaced persons along the China–Myanmar border: 2011–2014. *Malar J 15*: 471

57. Bradley J, Monti F, Rehman AM, Schwabe C, Vargas D, Garcia G, Hergott D, Riloha M, Kleinschmidt I., 2015. Infection importation: a key challenge to malaria elimination on Bioko Island, Equatorial Guinea. *Malar J 14*: 46

58. F. K. Darl; R. Bayoumi; T. AlKarm; ASFBMMH., 1993. Status of imported malaria in a control zone of the United Arab Emirates bordering an area of unstable malaria. *Trans R SCCIETY Trop Med Hyg 87*: 617–619

59. Kheang ST, Lin MA, Lwin S, Naing YH, Yarzar P, Kak N, Price T., 2018. Malaria Case Detection Among Mobile Populations and Migrant Workers in Myanmar: Comparison of 3 Service Delivery Approaches. *Glob Heal Sci Pract 6*: 384–389

60. Edwards HM, et al., 2015. Novel Cross-Border Approaches to Optimise Identification of Asymptomatic and Artemisinin-Resistant Plasmodium Infection in Mobile Populations Crossing Cambodian Borders. *PLoS One 10*: e0124300

61. Stratil A-S, Vernaeve L, Lopes S, Bourny Y, Mannion K, Hamade P, Roca-Feltrer A, Tibenderana JK, Sovannaroth S, Debackere M., 2021. Eliminating Plasmodium falciparum malaria: results from tailoring active case detection approaches to remote populations in forested border areas in north-eastern Cambodia. *Malar J 20*: 108

62. Li Z, et al., 2015. Malaria Imported from Ghana by Returning Gold Miners, China, 2013.

Emerg Infect Dis 21: 864–867

63. Tseroni M, et al., 2020. The Importance of an Active Case Detection (ACD) Programme for Malaria among Migrants from Malaria Endemic Countries: The Greek Experience in a Receptive and Vulnerable Area. *Int J Environ Res Public Health 17*: 4080
